# The Hospital System in Ireland

**Published:** 1898-10-15

**Authors:** Garrett Horder


### THE HOSPITAL SYSTEM IN IRELAND.

By Garrett Horder, L.R.C.P., M.R.C.S.

A good deal has been written about the necessity of reform in our metropolitan and provincial hospitals, and there is evidence that public interest has at last been fairly aroused.

From my investigations I have come to the conclusion that there is quite as much, if not more, necessity for reforms in the medical charities of Ireland. I purpose briefly to give an outline of the hospital system in the principal towns of that country. In Ireland is to be found a system altogether different from any existing either in England or Scotland. In the first place each county has its own infirmary, and these infirmaries are almost entirely supported by rates levied on the landowners and tenants of land and houses. Up to the passing of the new Local Government Bill it was the duty of the grand jury at each assize to make a "presentment"—*i.e.*, a claim was presented for the amount that would be required for the support or maintenance of a county infirmary for a period (I imagine) of half a year. According to an old Act of Parliament donors of a sum of thirty guineas, and subscribers of a sum of not less than three guineas annually, were constituted governors of the infirmary. With very few exceptions these infirmaries have paid surgeons appointed to them; and many keen struggles have taken place in the past to obtain these much coveted appointments. The Irish Law Reports furnish evidence of many disputes over the election of such officers.

Mention has been made of donors and subscribers, but practically the amount subscribed is very small indeed. I have not the figures at hand, but as far as my memory serves me the total income from all sources of these county infirmaries in the year 1891 amounted to about £26,000, and the amount received from the County "Cess" came to no less than £22,000. Under the new Local Government Act the County Councils will have to raise the money, and will very properly have a proper proportion of the representation on the managing committees. It will be noted that practically speaking the county infirmaries of Ireland are rate-supported institutions. From what I have been able to gather, the tax is an uncommonly light one; and as the small farmers and their families make use of these infirmaries to a large extent, it follows that the small holders get more than a *quid pro quo* for the money they have to pay. Turning our attention to Dublin, we find that many of the hospitals are not only municipalised, but are also subsidised by the Government. Last year 11 hospitals received grants from the Government amounting to no less a sum than £16,022! Seven out of the 11 hospitals received £1,750 from the Corporation of Dublin; and another hospital (the Meath) received £1,000 from the county of Dublin. Another rather startling fact about the same hospitals is that patients paid a sum amounting to £7,895 towards their maintenance. The total income from every source of the same hospitals amounted to £48,969, of which amount only £6,574 was derived from subscriptions and donations. The total number of patients treated in these hospitals was 11,981. I am not in a position to state accurately the number of out or dispensary patients treated, because the hospital authorities unfortunately give in their reports only the total number of attendances made by that class of patients. It is to be hoped that they (the managers) will adopt the same plan that is carried out in England and Scotland, and register the individual patients and not the number of times they attend the hospitals.

All the hospitals referred to are visited and examined periodically by the Board of Superintendence; some are also visited and examined by two other committees, viz., the Committee of the Dublin Corporation and the committee appointed by the Council of the Hospital Sunday Fund. It is therefore evident that Dublin is far ahead of our own metropolis with respect to the interest which is taken in her medical charities. I may add that all these committees draw up reports, and publish them for the use of the public. In addition to the 11 hospitals, Dublin possesses no less than 20 other hospitals with 1,464 beds. In 15 of these hospitals 10,697 patients were treated last year (1897). In the other five the returns are not available. Adding these figures to those already given we find that 22,678 patients were admitted during the year 1897. Taking the population of greater Dublin to be as stated by the Medical Officer of Meath (349,594) it follows that one person in every 16 of the population was an in-patient of one of the Dublin hospitals. I hesitate to say what the proportion of out-patients to the population really was for the reason already assigned, but it is perfectly evident that the proportion was very high. It may here be noted that the Council of the Dublin Hospital Sunday Fund take no note of ordinary out-patients in making their distribution of money. They do, however, take into consideration the number of accidents treated in the extern department, and also the number of maternity cases attended outside the hospitals. As there has always been a good deal of mystery attached to the mode of awarding grants to the London hospitals by the Hospital Sunday Fund Council, it may be as well to mention that the Dublin Hospital Sunday Fund Council publish figures showing exactly how they arrange their awards.

In the investigation of the Dublin hospitals, the following points have occurred to me as being worthy of observation: (1) The large number of hospitals in proportion to the population; (2) the large amount paid by patients towards their maintenance; (3) the large number of infectious diseases treated in general hospitals; and (4) the large amount subscribed by Government towards the maintenance of certain hospitals.

